# Association of preadmission metformin use and prognosis in patients with sepsis with diabetes: a systematic review and meta-analysis

**DOI:** 10.3389/fendo.2026.1815219

**Published:** 2026-04-20

**Authors:** Mingying Zhang, Zhibin Lin, Chao Chen, Xiaoze Zhong, Weijun Liu, Zhanmao Chen, Tietao Chen, Chengqing Song, Guanyuan Tian, Kefei Wu

**Affiliations:** 1 Department of Nephrology, Jieyang People’s Hospital, Jieyang, China; 2 Jieyang Medical Research Center, Jieyang People’s Hospital, Jieyang, China

**Keywords:** diabetes, metformin, mortality, sepsis, systematic review with meta-analysis

## Abstract

**Background:**

Preadmission metformin may lower mortality in diabetic sepsis patients, but evidence is conflicting, necessitating a systematic review and meta-analysis for confirmation.

**Methods:**

We systematically searched MEDLINE (via PubMed), EMBASE, and Cochrane CENTRAL from inception to September 1, 2025, for cohort studies evaluating metformin use in septic patients with diabetes. Study quality was assessed using the Newcastle–Ottawa Scale. Two reviewers independently screened studies, extracted data, and evaluated methodological quality. Meta-analysis was conducted using STATA statistical software and Review Manager software, calculating pooled odds ratios with 95% confidence intervals via the inverse variance random-effects model. The MET group included diabetic sepsis patients with preadmission metformin exposure, and the NM group included those without.

**Results:**

This meta-analysis of 14 studies (12,687 patients), all with low bias risk, demonstrated that preadmission metformin use in sepsis-diabetes patients was associated with reduced overall mortality (OR 0.58, 95% CI 0.44–0.75, P < 0.00001). Significant reductions were observed in 28-day (OR 0.61, P = 0.002), 90-day (OR 0.48, P = 0.001), 365-day (OR 0.33, P = 0.0005), and in-hospital mortality (OR 0.43, P < 0.02). However, 30-day (OR 0.71, P = 0.06), 60-day (OR 0.72, P = 0.22), and ICU mortality (OR 0.76, P = 0.25) showed no significant differences. Notably, metformin also significantly improved serum creatinine (MD −0.32, P = 0.04) and metformin usage was associated with elevated serum lactate levels.

**Conclusions:**

This meta-analysis links preadmission metformin use in diabetic sepsis patients to reduced mortality—particularly 28-day, 90-day, 365-day, and in-hospital—along with decreased serum creatinine. Clinically and from a public health standpoint, these data support the integration of metformin history as a favorable prognostic indicator into updated clinical guidelines, thereby informing future antimicrobial stewardship and sepsis bundle strategies. These findings support further evaluation of metformin’s benefits in large-scale, multicenter randomized controlled trials.

## Introduction

Sepsis is a life-threatening organ dysfunction caused by a dysregulated host response to infection, and it remains one of the leading causes of mortality among patients in intensive care units (ICUs) worldwide (1). In recent years, the incidence of sepsis has continued to rise, affecting tens of millions of people globally each year and resulting in millions of deaths, particularly placing a heavy disease burden on low- and middle-income countries (2, 3). The pathophysiology of sepsis is complex, involving initial overactivation of the immune system followed by immunosuppression, leading to cellular metabolic disturbances, microcirculatory dysfunction, and multiple organ failure (4). Despite significant advances in antimicrobial therapy, organ support technologies, and critical care, the mortality rate of septic patients remains high, with severe cases reaching 30% to 50%, underscoring the urgent need to explore effective adjuvant treatment strategies (5, 6).

In 2024, an estimated 589 million adults aged 20–79 years are living with diabetes worldwide (7). China accounts for the highest global burden, a situation driven by its aging population, rising rates of overweight and obesity, and shifts toward unhealthy lifestyles (7). Diabetic patients, as a special population, are more susceptible to severe infections due to their immune dysfunction and chronic inflammatory state (8, 9). Studies have shown that the risk of developing sepsis is approximately 30% to 40% higher in diabetic patients compared to non-diabetic individuals, with more complications and poorer prognoses following infection (10, 11). Hyperglycemic conditions promote the growth of pathogenic microorganisms, while diabetes is often accompanied by vascular complications, neuropathy, and impaired tissue repair ability. These factors collectively contribute to a higher likelihood of infection progressing to sepsis and an increased mortality risk among diabetic patients (12, 13). Patients with sepsis with diabetes often require longer hospital stays, more medical resources, and higher healthcare costs, posing significant challenges to healthcare systems and socioeconomic stability.

Metformin is the most widely used first-line oral hypoglycemic agent globally. Compared with traditional sulfonylureas, it offers advantages such as a lower risk of hypoglycemia, no weight gain (or even weight loss assistance), and cardiovascular protective effects. It is recommended as an initial treatment for type 2 diabetes(T2D) by multiple international guidelines (7, 14, 15). The drug exerts its glucose-lowering effects through multiple mechanisms. Studies have found that metformin can activate the AMP-activated protein kinase (AMPK) pathway, inhibit mammalian target of rapamycin (mTOR) signaling, and modulate autophagy and mitochondrial function, thereby exerting anti-inflammatory, antioxidant, and immunomodulatory effects (16–20). These immunomodulatory properties are particularly significant for sepsis, a disease characterized by immune dysregulation. A large retrospective cohort study of diabetic patients without CKD hospitalized for COVID-19 found that preadmission metformin use was associated with a significantly lower risk of in-hospital mortality and other severe outcomes (21). Therefore, theoretically, metformin may exert protective effects in septic patients through multiple pathways, such as mitigating the inflammatory storm, improving immune cell function, and inhibiting pathogen proliferation (22).

Despite the aforementioned encouraging laboratory findings and observational data, there remains significant inconsistency and controversy regarding the impact of pre-admission metformin use on clinical outcomes in septic patients with diabetes. Some studies suggest that pre-admission metformin use can significantly reduce mortality in septic patients with diabetes. The study by YANG et al. (23). demonstrated that pre-admission metformin use was associated with a 39% reduction in 30-day mortality. Additionally, patients not taking metformin had a 2.5 times higher risk of death within 28 days compared to those taking metformin (24). The body of evidence from these meta-analyses indicates that preadmission metformin is linked to reduced mortality and is safe in patients with sepsis and diabetes, providing a rationale for its assessment in subsequent trials (25, 26). However, other studies indicate that among septic patients with diabetes, metformin use is not associated with altered sepsis outcomes or host response (27, 28). Therefore, it is crucial to synthesize current evidence to determine the efficacy of metformin in enhancing clinical outcomes for diabetic patients with sepsis.

## Methods

### Search strategy

**Figure 1:**
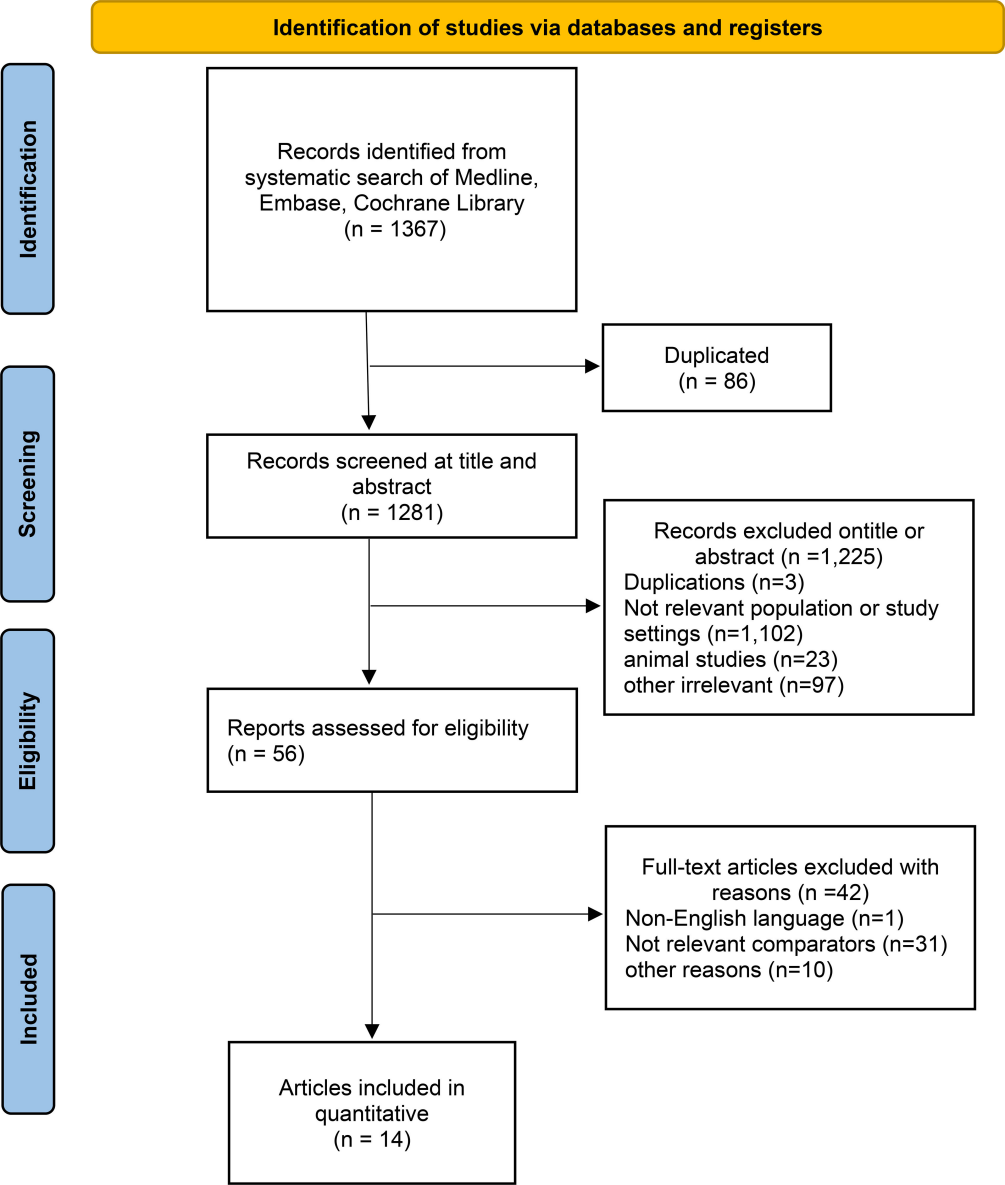
PRISMA 2020 flow diagram for new systematic reviews which included searches of databases and registers only.

This systematic review and meta-analysis was reported following the updated Preferred Reporting Items for Systematic Reviews and Meta-Analyses (PRISMA) (29) and Meta-analysis of Observational Studies in Epidemiology (MOOSE) guidelines (30). The study protocol was registered in International Prospective Register of Systematic Reviews (PROSPERO) on 10 April, 2023 (CRD42023412696). The PRISMA 2020 checklist is shown as **Supplementary Table 1**, the study profile is presented in **Figure 1**.

### Flowchart of the study selection process

A systematic literature search was conducted in the MEDLINE databases (via the PubMed search engine), Cochrane, and Embase databases for articles published in English prior to September 1, 2025. To ensure the feasibility of the search strategy and the completion of comprehensive data analysis within project resource constraints (time, funding), we prioritized major international English-language databases. Search strategies incorporated controlled vocabulary (MeSH and Emtree) as well as free-text terms in titles, abstracts, and keywords. The key concepts explored included “metformin”, “sepsis”, and “critically ill”. Literature screening was performed using EndNote X9. Additionally, the reference lists of included studies were examined to identify further relevant publications. Furthermore, a manual search was conducted on grey literature, preprint servers, conference abstracts, and reference lists to uncover potentially relevant studies as well as related reviews and editorials. Two independent researchers carried out the literature retrieval process. The detailed search strategy is provided in **Supplementary Table 2**.

### Eligibility criteria

Our inclusion criteria were strictly defined using the PICOS (Population, Intervention, Comparator, Outcomes, and Study design) framework to minimize clinical and metabolic heterogeneity: Population (P): Adult patients (≥ 18 years old) with a pre-existing diagnosis of diabetes mellitus who were admitted with a diagnosis of sepsis. Intervention/Exposure (I): Preadmission or pre-illness use of metformin. Comparator (C): Septic patients with diabetes who were not using metformin prior to admission. To address potential uncontrolled confounding, studies that utilized non-diabetic septic patients as the comparative group were explicitly excluded. Outcomes (O): The primary outcomes were reported mortality rates (e.g., 28-day, 90-day, 365-day, or in-hospital mortality) comparing metformin users and non-users. Study design (S): Observational studies published in English and conducted in any clinical setting (emergency department, hospital ward, or intensive care unit). Exclusions were applied to studies that did not report relevant outcome data, focused on mortality in sepsis compounded by specific additional severe comorbidities, lacked full-text availability, or were non-original publications such as commentaries, reviews, or editorials.

### Eligible studies and extracted data

Two reviewers independently assessed the eligibility of studies and extracted relevant data. Any discrepancies between their assessments were resolved through discussion until a consensus was reached, with involvement of a third author when necessary. The following information was collected from each included study: first author, year of publication, study design, setting (single or multicenter), sample size, study period, sex distribution and mean age of participants across groups, and primary outcome measures.

### Risk of bias evaluation

The risk of bias in the included cohort studies was evaluated using the Newcastle–Ottawa Scale (NOS). The NOS rates studies across three domains: cohort selection (maximum of 4 points), comparability of cohorts (maximum of 2 points), and outcome assessment (maximum of 3 points), for a total possible score of 9. Studies scoring between 7 and 9 points were deemed to be of high quality and at low risk of bias (31). The risk of bias was independently assessed by two reviewers using the ROBINS-I tool, which evaluates seven bias domains and categorizes overall risk as low, moderate, serious, or critical. Any discrepancies were resolved through discussion. The quality of evidence for outcomes was evaluated using the GRADE approach, which classifies evidence into four levels (high, moderate, low, or very low). The quality of evidence from the included observational studies was assessed as ‘low’ initially and adjusted based on standard GRADE criteria.

### Statistical analysis

The primary outcomes of interest were mortality metrics among diabetic patients with sepsis, comparing preadmission metformin users to non-users. Meta-analyses were conducted to pool the effect estimates, calculating summary odds ratios (ORs) and corresponding 95% confidence intervals (CIs) from individual studies.

Between-study heterogeneity was assessed using the Chi-square (χ²) test and quantified by the I² statistic. An I² value > 50% or a P-value ≤ 0.10 was considered indicative of substantial heterogeneity among the included studies. In such cases, a random-effects model was adopted to calculate the pooled statistics, and further analyses were conducted to explore the sources of heterogeneity. Conversely, if heterogeneity was low, a fixed-effect model was applied.

Potential publication bias was initially evaluated through visual inspection of funnel plot asymmetry. To quantitatively confirm these visual findings and ensure methodological rigor, both Begg’s rank correlation test and Egger’s linear regression test were performed (32). A P-value < 0.10 in either test was considered suggestive of small-study effects or significant publication bias. All statistical analyses were conducted using STATA software (version 15.0, StataCorp LLC, College Station, TX, USA) and Review Manager (RevMan version 5.3, The Cochrane Collaboration).

Finally, the certainty of evidence for each outcome was assessed using the GRADE (Grading of Recommendations Assessment, Development and Evaluation) approach. The overall quality of evidence was classified as high, moderate, low, or very low based on systematic evaluations of study design, risk of bias, inconsistency, indirectness, imprecision, and publication bias.

## Results

### Study selection

A total of 1,367 records were initially identified through the search strategy. A total of 86 duplicate entries were removed, and 1,281 records were screened based on titles and abstracts. After screening titles and abstracts, 56 cohort studies were deemed potentially relevant to the research objective. Upon full-text review and application of inclusion criteria, 14 studies comprising a total of 12,687 patients were included in the final meta-analysis. Although our search strategy included preprint servers, all 14 studies ultimately included in this meta-analysis were published in peer-reviewed journals.

### Study characteristics

All studies included in the analysis were observational cohort investigations involving septic patients with diabetes who received metformin treatment prior to hospitalization (23–25, 27, 28, 33–41). Among these, three studies (24, 25, 37) reported 28-day mortality, five studies (23, 27, 28, 35, 40) provided data on 30-day mortality, one study (27) reported 60-day mortality, four studies (27, 39–41) reported 90-day mortality, two studies (40, 41) reported 1-year mortality, three studies (27, 38, 40) documented ICU mortality, and four studies (27, 36, 38, 41) recorded hospital mortality. Primary outcome measures were systematically extracted from each study. In cases where odds ratios (ORs) and 95% confidence intervals (CIs) were not directly reported, these values were calculated using the raw data available in the original articles. The baseline characteristics of the included studies are summarized in **Supplementary Table 3**.

### Risk of bias evaluation

The analyzed literature comprised twelve retrospective cohort studies and two prospective observational study. According to the Newcastle–Ottawa Scale (NOS), all included studies received a score of six or higher, suggesting a low overall risk of bias. A detailed breakdown of the risk of bias assessments is available in **Supplementary Table 4**. ROBINS-I assessments showed that all studies were judged to have a low overall risk of bias (**Supplementary Figure 4**).

### Confidence of evidence

The certainty of evidence for the association between preadmission metformin use and mortality outcomes (28-day, 90-day, 365-day, and in-hospital mortality) was assessed using the GRADE framework. Given that all included studies were observational in design, the evidence was initially graded as low. Although the risk of bias was low and no significant publication bias was detected, the certainty of the evidence remained Low due to the observational nature of the data. No factors warranting an upgrade (e.g., large magnitude of effect) were consistently present to justify raising the certainty level. The detailed GRADE evidence profile is presented in **Supplementary Table 5**.

### Effects of metformin on outcomes

**Figure 2:**
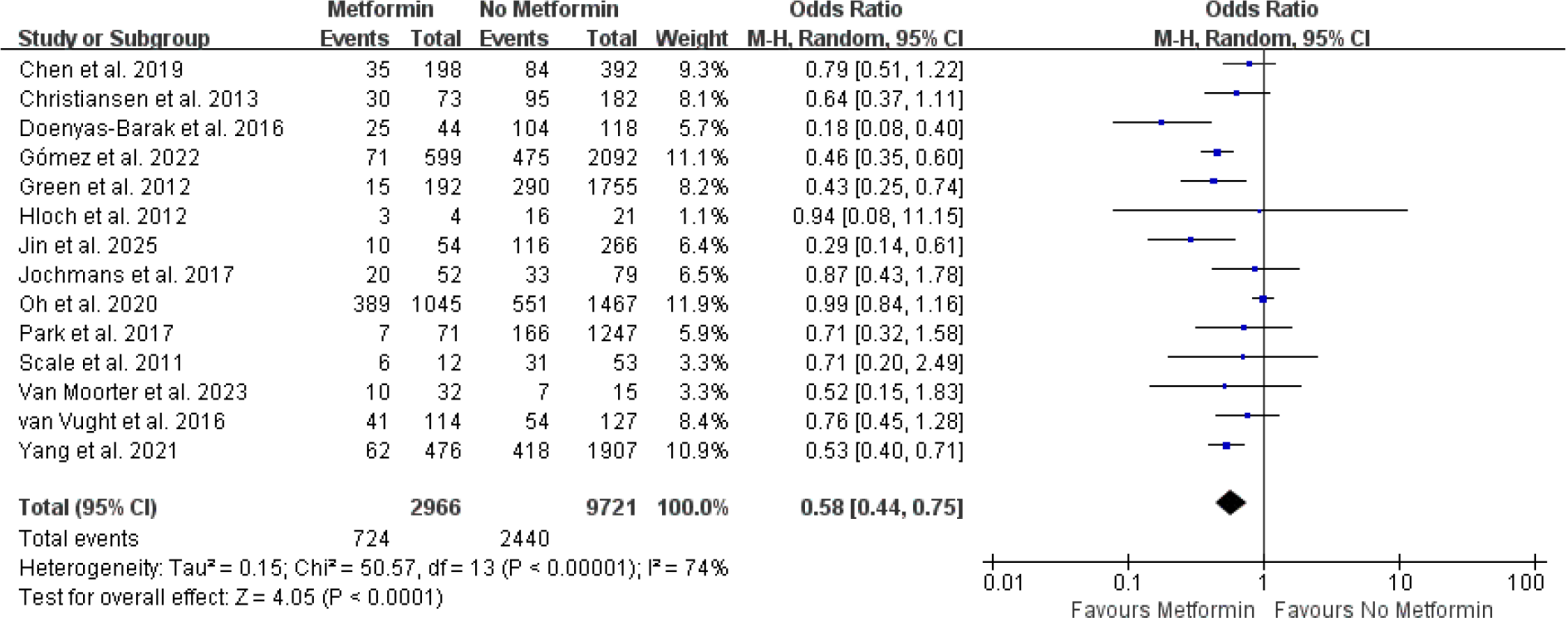
Meta-analysis of the overall pooled ORs of studies investigating the mortality outcomes of patients with sepsis with diabetes. The Forest plot shows the significance of the association between metformin use and mortality in patients with sepsis with diabetes according to the random effects model.

Among the 14 included cohort studies (comprising 12,687 patients), all incorporated studies reported mortality outcomes, demonstrating moderate heterogeneity (I² = 74.0%, P < 0.00001). The results of the meta-analysis with a random-effects model showed that septic patients who received metformin prior to hospitalization had significantly lower mortality compared to those without metformin use (OR, 0.58; 95% CI, 0.44–0.75, P < 0.00001) (**Figure 2**) (23–25, 27, 28, 33–41).

**Figure 3:**
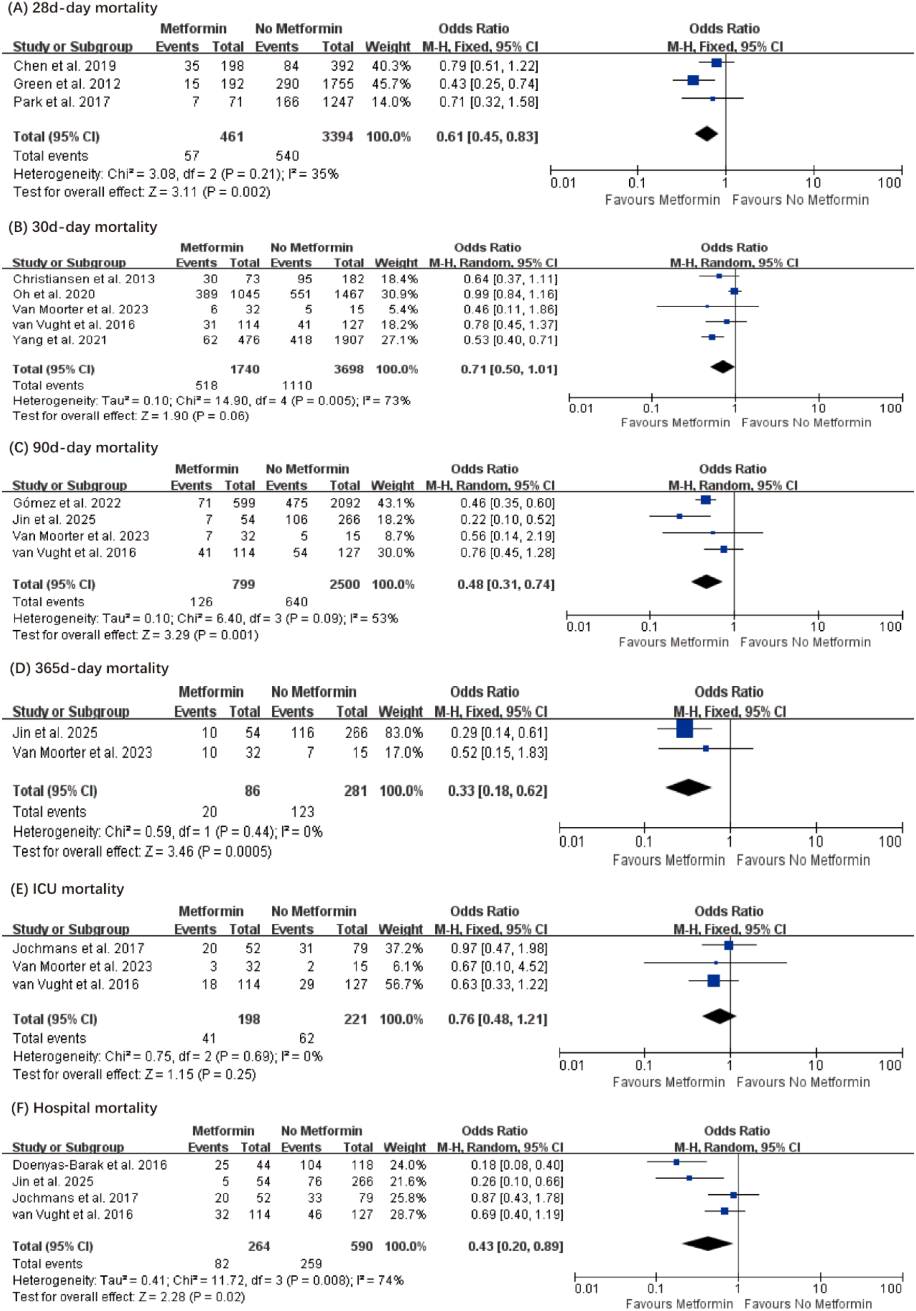
Subgroup meta-analysis of the overall pooled ORs of studies investigating the mortality outcomes in patients with sepsis and diabetes. The forest plot demonstrates the association between metformin use and 28-day mortality, 90-day mortality, 365-day mortality, as well as in-hospital mortality in patients with sepsis and diabetes.

Subsequently, we performed subgroup analyses based on discrepancies in reported mortality rates among the studies (**Figure 3**). The subgroup analysis under the random-effects model showed that, compared with non-metformin users, septic patients receiving preadmission metformin had significantly lower 90-day mortality (OR, 0.48; 95% CI, 0.48–0.74, P = 0.001) (27, 39–41), 365-day mortality (OR, 0.33; 95% CI, 0.18–0.62, P = 0.0005) (40, 41), and in-hospital mortality (OR, 0.43; 95% CI, 0.20–0.89, P < 0.02) (27, 36, 38, 41). Under the fixed-effect model, 28-day mortality was also significantly reduced in the metformin group (OR, 0.61; 95% CI, 0.45–0.83, P = 0.002) (24, 25, 37). Heterogeneity decreased slightly after subgroup analysis compared to the pre-subgroup results, with no detected heterogeneity (I² = 35.0%, P = 0.21), moderate heterogeneity (I² = 53.0%, P = 0.09), no detected heterogeneity (I² = 0%, P = 0.44), and moderate heterogeneity (I² = 74.0%, P = 0.008), respectively. Furthermore, subgroup analyses of 30-day mortality, 60-day mortality, and ICU mortality showed no significant differences between the metformin and non-metformin groups: 30-day mortality (OR, 0.71; 95% CI, 0.50–1.01, P = 0.06) (23, 27, 28, 35, 40), 60-day mortality (95% CI, 0.43–1.22, P = 0.22) (27), and ICU mortality (OR, 0.76; 95% CI, 0.48–1.21, P = 0.25) (27, 38, 40).

**Figure 4:**
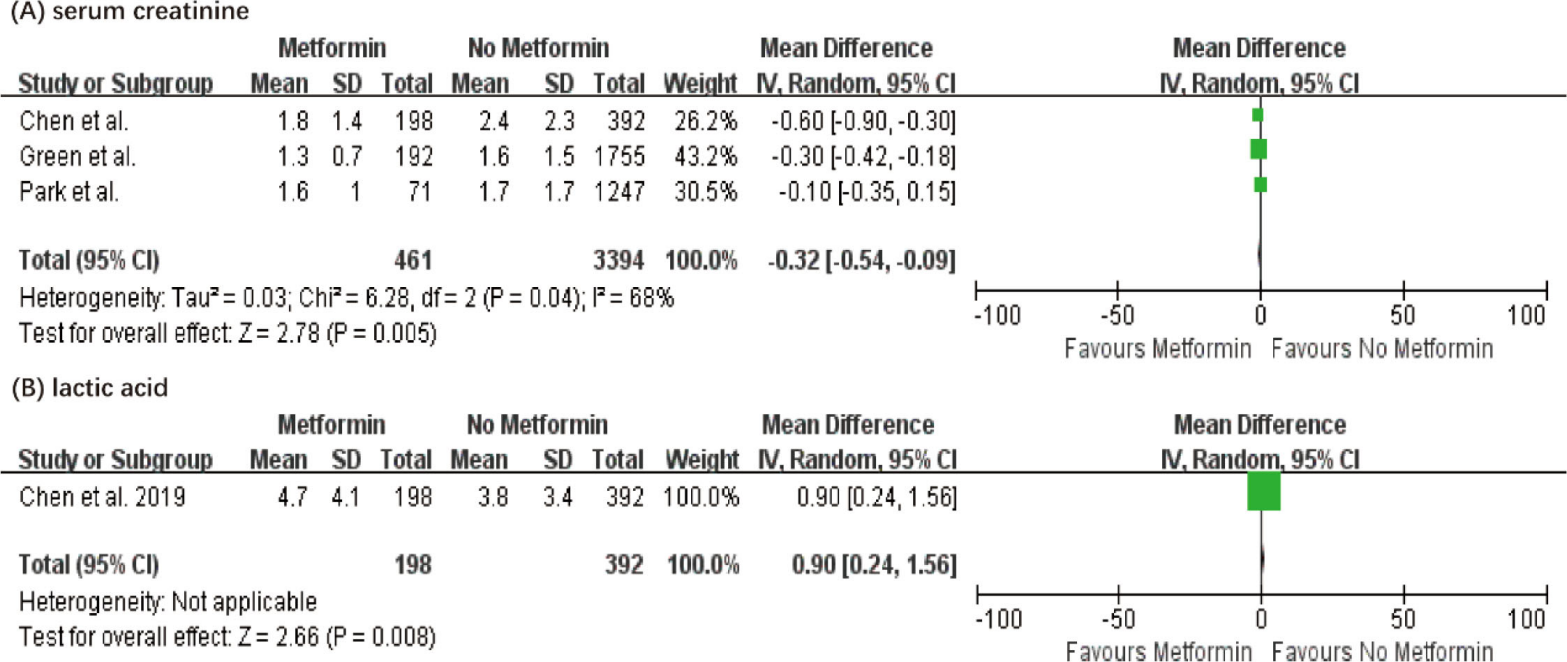
The Forest plot demonstrates the association between preadmission metformin use and serum creatinine and lactate levels in patients with sepsis and diabetes.

Surprisingly, metformin also showed a significant greater improvement in the levels of serum creatinine (MD = −0.32, 95% CI = −0.35 to −0.09, P = 0.04). Notably, patients in the metformin group exhibited significantly higher serum lactate levels compared to the non-metformin group (MD 0.90, 95% CI 0.24–1.56, P = 0.008), which warrants careful clinical monitoring. (**Figure 4**).

**Figure 5:**
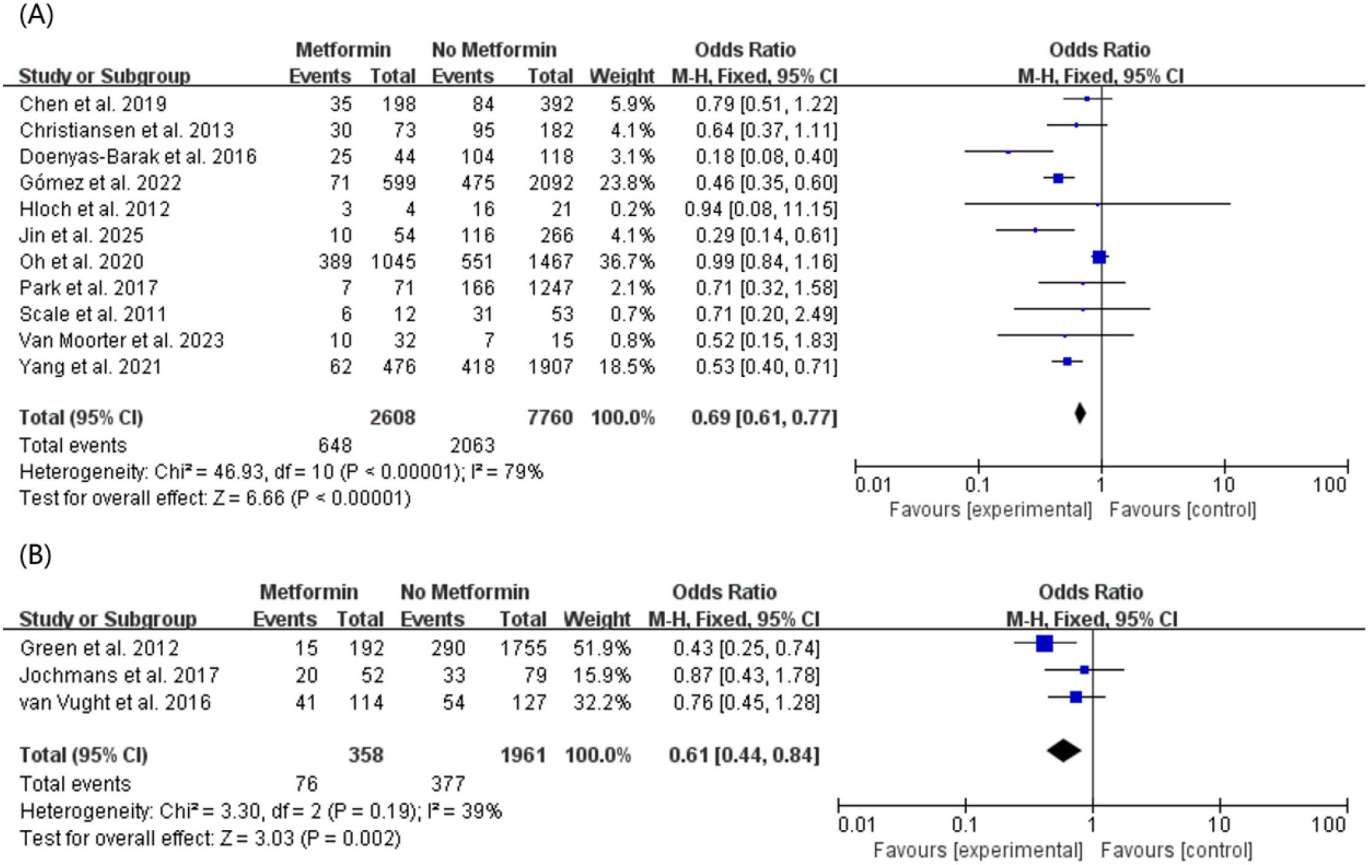
Subgroup meta-analysis of mortality outcomes stratified by diabetes classification. The forest plot demonstrates the association between preadmission metformin use and mortality in patients with confirmed Type 2 Diabetes Mellitus (T2DM) and those with generalized diabetes mellitus (unspecified T1DM or T2DM).

Furthermore, to clarify the applicable population and address the pathophysiological differences between diabetes classifications, we conducted a subgroup analysis based on the specific type of diabetes reported (**Figure 5**). Among the included literature, 11 studies explicitly restricted their cohorts to patients with confirmed Type 2 Diabetes Mellitus (T2DM). The pooled results for this specific subgroup robustly demonstrated that preadmission metformin use was significantly associated with lower mortality in sepsis patients with T2DM (OR, 0.69; 95% CI, 0.61–0.77, P < 0.00001) (**Figure 5A**). The remaining 3 studies, which utilized a generalized “Diabetes Mellitus” classification encompassing all diabetes patients, also showed a significant reduction in mortality (OR, 0.61; 95% CI, 0.44–0.84, P = 0.002) (**Figure 5B**). These findings confirm that the prognostic benefits of metformin are highly applicable to the T2DM patient population.

**Figure 6:**
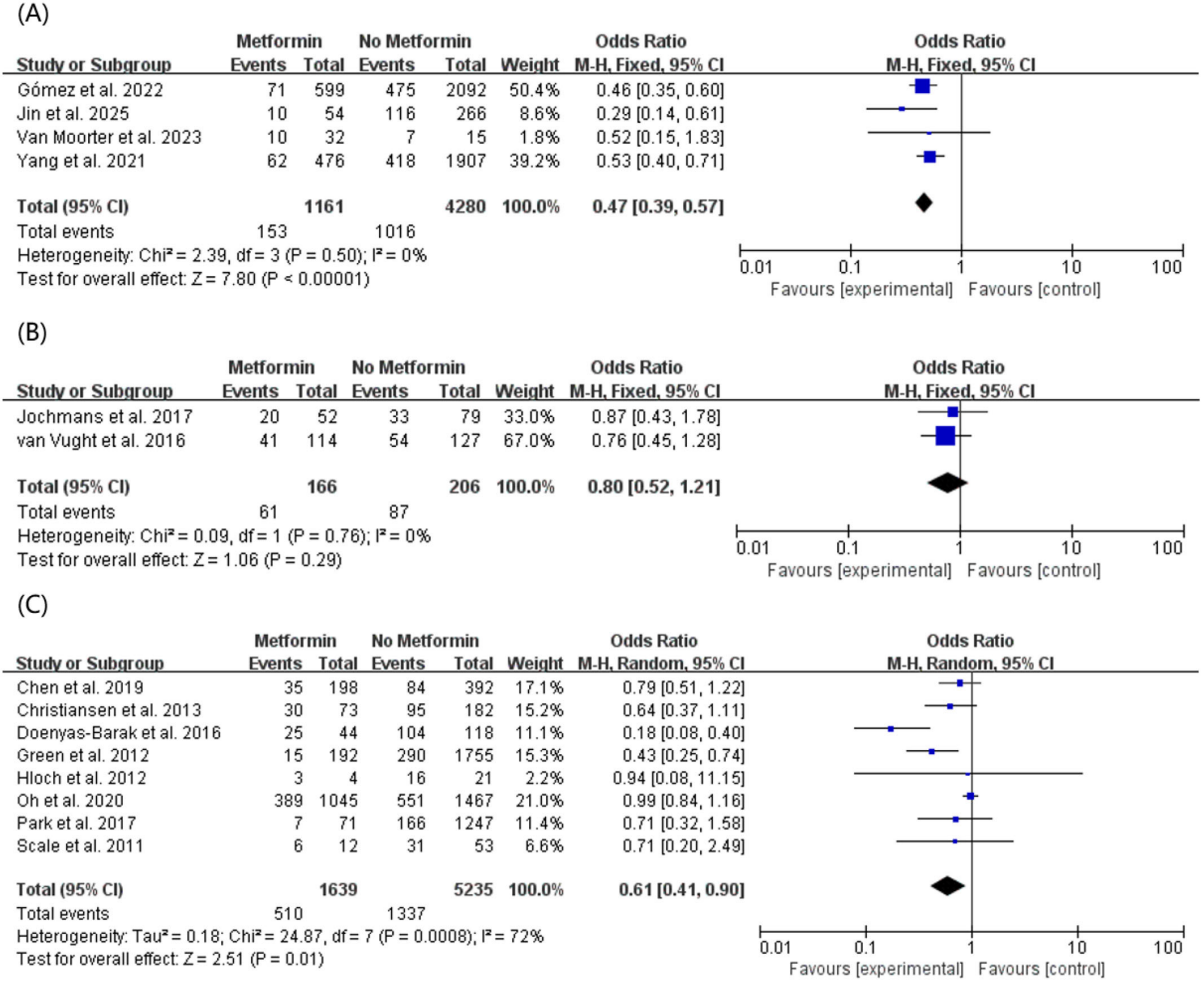
Subgroup meta-analysis of mortality outcomes stratified by sepsis diagnostic criteria. The forest plots demonstrate the association between preadmission metformin use and mortality in patients diagnosed according to **(A)** Sepsis-3 (SOFA-based) criteria, **(B)** Sepsis-2 criteria, and **(C)** Sepsis-1/2 (SIRS-based) criteria.

To assess the impact of criterion heterogeneity on the combined effect size, we stratified the studies based on the clinical diagnostic criteria for sepsis (**Figure 6**). Among the included literature, 8 studies defined sepsis based on Sepsis-1/2 (SIRS-based) criteria, 2 studies utilized strict Sepsis-2 criteria, and 4 studies adopted the updated Sepsis-3 (SOFA-based) criteria. The pooled results demonstrated a robust and highly significant survival benefit in patients diagnosed under Sepsis-3 criteria (OR, 0.47; 95% CI, 0.39–0.57, P < 0.00001) (**Figure 6A**) and Sepsis-1/2 (OR, 0.61; 95% CI, 0.41–0.90, P = 0.0008) (**Figure 6C**). However, in the subgroup utilizing Sepsis-2 criteria, the protective effect of metformin was attenuated and did not reach statistical significance (OR, 0.80; 95% CI, 0.52–1.21, P = 0.29) (**Figure 6B**).

### analyse Sensitivity s

**Figure 7:**
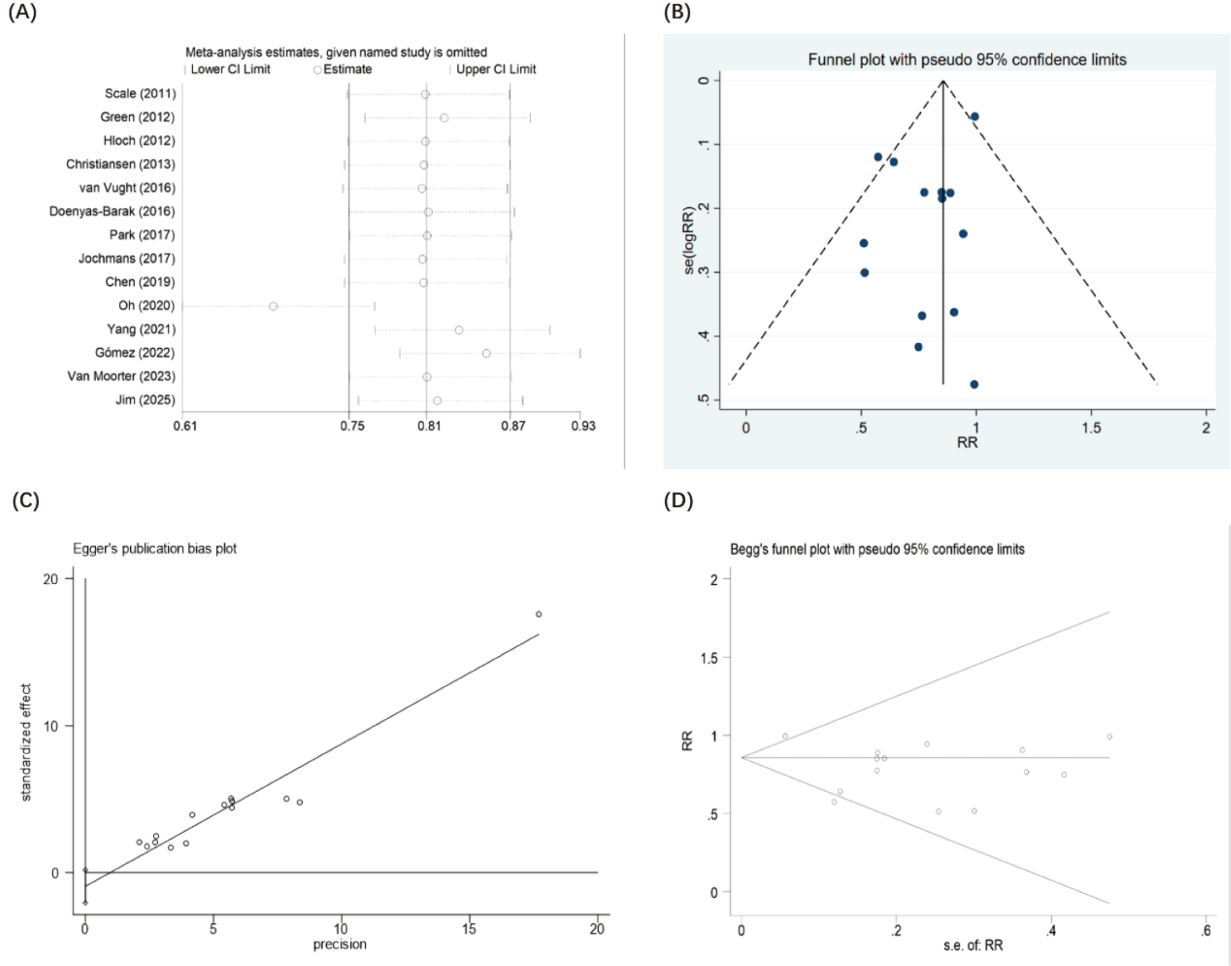
Sensitivity analysis, which involved systematically excluding each study one by one and recalculating the combined effect estimate for the remaining studies, confirmed that the findings on metformin use and mortality in septic patients with diabetes were robust and reliable **(A)**. Funnel plot evaluating mortality after the preadmission use of metformin in septic patients with diabetes **(B)**. Egger’s funnel plot evaluating mortality after the preadmission use of metformin in septic patients with diabetes **(C)**. Begg’s funnel plot evaluating mortality after the preadmission use of metformin in septic patients with diabetes **(D)**.

Given that all included studies were observational cohorts with minimal risk of bias (**Supplementary Table 4**), no sensitivity analysis was carried out based on methodological criteria. Instead, sensitivity analysis was exclusively applied to evaluate the impact of each individual study on the overall odds ratio and 95% confidence interval by sequentially removing one study per iteration. The outcomes confirmed that the aggregated results remained consistent and reliable (**Figure 7A**). Furthermore, when metformin users were categorized according to clinical outcomes, no notable heterogeneity was observed within any of the subgroups (**Supplementary Figure 1**).

### Evaluation of publication bias

We comprehensively evaluated potential publication bias regarding the primary mortality outcome and its respective subgroups. Initial visual inspection of the funnel plot for overall mortality revealed a generally symmetrical distribution of the effect sizes, suggesting an absence of substantial publication bias (**Figure 7B**).

To quantitatively confirm this visual assessment, both Egger’s linear regression test (**Figure 7C**) and Begg’s rank correlation test (**Figure 7D**) were performed on the overall mortality data. The statistical results corroborated the visual findings, indicating no significant small-study effects or publication bias (Egger’s test: *P* = 0.095; Begg’s test: *P* = 0.511).

Furthermore, we assessed publication bias within the specific mortality subgroups. Quantitative testing demonstrated no significant publication bias for 28-day, 90-day, and in-hospital mortality using Egger’s test (*P* = 0.722, 0.970, and 0.390, respectively; **Supplementary Figure 2**). (Note: Egger’s test could not be computed for the 365-day subgroup due to the limited number of included studies). However, Begg’s test confirmed the absence of publication bias across all four subgroups, including 28-day, 90-day, 365-day, and in-hospital mortality (*P* = 1.000, 0.734, 1.000, and 1.000, respectively; **Supplementary Figure 3**). Collectively, these analyses confirm the robustness of the synthesized effect estimates.

## Discussion

This evidence-based meta-analysis included 12,687 patients and investigated whether metformin is associated with reduced mortality in septic patients with diabetes. The pooled results indicated that preadmission metformin use was associated with lower mortality compared to non-use in this population. Considering variations in endpoint definitions across studies, we performed subgroup analyses of mortality outcomes. The subgroup results demonstrated that, relative to non-metformin users, patients with preadmission metformin use had significantly lower 28-day mortality (OR, 0.61; 95% CI, 0.45–0.83, P = 0.002) (24, 25, 37), 90-day mortality (OR, 0.48; 95% CI, 0.48–0.74, P = 0.001) (27, 39–41), 365-day mortality (OR, 0.33; 95% CI, 0.18–0.62, P = 0.0005) (40, 41), and in-hospital mortality (OR, 0.43; 95% CI, 0.20–0.89, P < 0.02) (27, 36, 38, 41). Unfortunately, subgroup analyses of 30-day mortality, 60-day mortality, and ICU mortality showed no significant improvement with metformin use: 30-day mortality (OR, 0.71; 95% CI, 0.50–1.01, P = 0.06) (23, 27, 28, 35, 40), 60-day mortality (95% CI, 0.43–1.22, P = 0.22) (27), and ICU mortality (OR, 0.76; 95% CI, 0.48–1.21, P = 0.25) (27, 38, 40). The seemingly inconsistent findings of significant benefits at 28 and 90 days but not at 30 and 60 days are likely statistical artifacts resulting from the different sets of primary studies pooled in each subgroup. For instance, the 30-day subgroup was heavily influenced by specific large-scale cohorts, introducing substantial heterogeneity. Therefore, these time-point variations reflect inter-study methodological differences rather than a true biological fluctuation of metformin’s efficacy. Notably, the lack of significant improvement in ICU mortality (P = 0.25) suggests a crucial clinical boundary for this intervention. In the highest-acuity settings, the extreme physiological derangements and profound severity of illness may simply overwhelm the pleiotropic protective mechanisms of metformin. This indicates that while the drug aids overall survival in general ward settings, its pharmacological benefits might not be potent enough to reverse the trajectory of the ‘sickest of the sick’ who have already progressed to requiring intensive care. Therefore, our findings suggest that metformin may hold therapeutic potential for patients with sepsis and diabetes.

The association between metformin use and mortality in diabetic patients with infections remains a subject of ongoing debate in the scientific community. The available evidence on metformin use in septic patients shows conflicting results. While several studies (25, 27, 28, 34, 37) found no significant mortality differences between metformin users and non-users, other investigations specifically those by Yang et al. (23) and Gómez et al. (39) reported that preadmission metformin use was associated with significantly reduced mortality in this patient population. Subsequently, a meta-analysis incorporating five observational cohort studies (involving 1,282 patients) indicated that preadmission metformin use was associated with reduced mortality in patients with sepsis with diabetes (OR, 0.59; 95% CI, 0.43–0.79; P = 0.001). Subsequently, one meta-analysis incorporating five observational cohort studies (involving 1,282 patients) (25) and another analyzing 11 relevant studies (comprising 8,195 patients) (26) both demonstrated a consistent association between preadmission metformin use and reduced mortality in septic patients with diabetes. A recent large-scale study (28) revealed that preadmission metformin use was not significantly associated with the risk of sepsis or 30-day mortality in diabetic patients.

Notably, our analysis revealed a seemingly paradoxical finding: patients in the metformin group exhibited significantly higher serum lactate levels compared to the non-metformin group (MD 0.90, P = 0.008), yet experienced significantly lower mortality. In the conventional management of sepsis, hyperlactatemia is universally recognized as a hallmark of tissue hypoperfusion, microcirculatory failure, and impending refractory shock (Type A lactic acidosis). However, the relationship between higher lactate levels and reduced mortality in our study must be interpreted through a distinct pathophysiological and pharmacological lens.

The elevation in lactate levels among metformin users is fundamentally a metabolic byproduct of the drug’s mechanism of action, rather than a surrogate marker for worsening sepsis-induced tissue ischemia. Metformin exerts its effects primarily by partially inhibiting mitochondrial electron transport chain complex I, shifting cellular metabolism towards anaerobic glycolysis (Type B lactic acidosis). Furthermore, metformin inhibits hepatic gluconeogenesis, which is a primary physiological pathway for lactate clearance. Crucially, this metabolic shift is inherently coupled with cytoprotective mechanisms via AMPK activation, which mitigates the sepsis-induced cytokine storm and protects organ function (as evidenced by the significantly improved serum creatinine levels in the metformin group; MD -0.32, P = 0.04). Therefore, while close clinical monitoring for severe metformin-associated lactic acidosis (MALA) remains warranted, moderate hyperlactatemia in diabetic sepsis patients with prior metformin exposure should not be reflexively interpreted as clinical deterioration. Recognizing this “high lactate, low mortality” paradox is vital for clinicians to avoid inappropriate or aggressive clinical escalations.

It is important to acknowledge and interpret the moderate heterogeneity (I^2^ = 74.0%) observed in our overall mortality analysis. Given the limited number of included studies (n = 14), conducting a multi-variable meta-regression was not statistically viable due to the high risk of overfitting. Instead, we systematically explored the sources of this heterogeneity through comprehensive, clinically driven subgroup analyses. Our findings indicate that the overall heterogeneity is primarily driven by methodological and clinical variations across the primary literature. Specifically, the discrepancies in mortality follow-up timeframes (e.g., 28-day vs. 365-day), the evolution of baseline sepsis severity (reflected by the transition from SIRS-based Sepsis-1/2 to SOFA-based Sepsis-3 diagnostic criteria), and variations in diabetes classifications contributed to the statistical variance. Notably, when the data were stratified by specific follow-up times (**Figure 3**) and diagnostic criteria (**Figure 6**), the I^2^ statistic was significantly reduced within several key subgroups. Coupled with our robust leave-one-out sensitivity analysis, these findings confirm the reliability and consistency of the synthesized survival benefit of preadmission metformin, despite the inherent clinical heterogeneity of the pooled cohorts.

Furthermore, in septic shock patients with T2D, metformin administration within 48 hours was associated with lower 90-day and 365-day mortality (41). Thus, although conclusions remain controversial across existing studies, our meta-analysis provides more comprehensive and robust evidence indicating that metformin use may reduce mortality in patients with sepsis with diabetes, including 28-day mortality, 90-day mortality, 365-day mortality, and in-hospital mortality.

To date, the mechanisms by which metformin reduces mortality in patients with sepsis with diabetes remain incompletely understood. The potential mechanisms underlying its mortality reduction in patients with sepsis with diabetes primarily involve its pleiotropic pharmacological effects. As an AMPK activator, metformin’s core mechanism includes the activation of the AMPK signaling pathway, which inhibits NF-κB-mediated inflammatory signaling, significantly reduces levels of pro-inflammatory cytokines such as TNF-α and IL-6, and thereby mitigates systemic hyperinflammation and organ damage (42). In experimental sepsis models using adult mice, studies have demonstrated that metformin administration ameliorates sepsis-induced damage in multiple organs, including brain injury (42), liver injury (43, 44), and cognitive dysfunction associated with neuronal damage (45), primarily through activation of the AMPK pathway. Furthermore, in young murine models, metformin has been shown to alleviate acute lung injury and systemic inflammation during sepsis through inhibition of the S100A8/A9–NLRP3–IL-1β signaling axis (46). Thus, metformin can modulate immune cell function, ameliorate the immunosuppressive state in the late phase of sepsis, and protect vital organs such as the lungs, liver, and heart by regulating autophagy, reducing oxidative stress, and inhibiting cellular apoptosis. Furthermore, metformin helps restore gut microbiota balance, enhances intestinal barrier function, and reduces bacterial and endotoxin translocation. The synergistic effects of these multi-faceted mechanisms including anti-inflammation, immunomodulation, organ protection, and metabolic regulation may form the critical foundation for its ability to improve clinical outcomes in sepsis. However, the exact mechanisms still require further validation through high-quality studies.

Our study reveals that preadmission metformin use is associated with significantly reduced mortality in diabetic patients with sepsis. This finding holds direct clinical relevance, suggesting that rather than routine discontinuation, metformin therapy should be actively considered for continuation upon hospital admission after careful patient assessment. Furthermore, diabetes educators and clinical pharmacists can use this evidence to educate patients about metformin’s potential pleiotropic benefits, thereby reinforcing the importance of medication adherence. From a public health perspective, this research extends metformin’s potential applicability from diabetes management to sepsis prevention and care, providing relevant evidence for updating both sepsis bundle protocols and diabetes clinical guidelines. These findings illuminate that integrating optimized chronic disease management into the acute infection response framework represents a promising public health strategy.

### Limitations

Although the findings of our meta-analysis are encouraging, evidence regarding whether metformin can improve survival in septic patients with diabetes remains preliminary. This study has several limitations. First, the number of patients included in each study was relatively small, particularly after subgroup analyses were performed, which may have resulted in insufficient statistical power to accurately evaluate intergroup differences. This should be carefully considered when interpreting the pooled results. Second, all included studies were observational in design, and no randomized controlled trials (RCTs) were incorporated. Consequently, we can only establish an association between preadmission metformin use and reduced mortality, not definitive causation. A substantial risk of “healthy user bias” exists; patients consistently prescribed and tolerating metformin may inherently possess better baseline renal function, superior medication adherence, or more favorable socioeconomic backgrounds compared to those relying on insulin or other agents. This unmeasured confounding cannot be fully mitigated. Third, most original studies lacked granular reporting on specific daily dosages, the total duration of preadmission metformin therapy, and crucially, whether metformin was continued or suspended upon hospital admission. In-hospital continuation versus discontinuation represents a major clinical confounding variable that this study could not account for, making it impossible to ascertain an ‘effective exposure threshold’ or dose-response relationship. Fourth, the current meta-analysis is inherently constrained by the lack of granular clinical data within the primary observational studies. For instance, evaluating the efficacy of metformin across different sepsis severity tiers was precluded because only isolated studies reported specific clinical scoring systems (e.g., APACHE II (36, 37), SOFA (37), qSOFA (25), or SAPS II (23)). Furthermore, most original studies did not report patient-level data regarding specific daily dosages, the total duration of preadmission metformin therapy, or medication compliance. Similarly, many database-driven studies relied on broad diagnostic codes (e.g., ICD codes) rather than explicit biochemical cutoffs to define exclusion criteria, such as specific eGFR thresholds for “severe renal dysfunction” or explicit transaminase levels for “liver dysfunction.” This reliance on aggregate, retrospectively coded data makes it currently impossible to establish an “effective exposure threshold,” determine a dose-response relationship, or standardize clinical exclusion criteria. These represent shared limitations of the existing literature that future prospective cohorts or individual patient data (IPD) meta-analyses must address to minimize selection bias.

Furthermore, a critical limitation of our meta-analysis—and inherently of the underlying observational literature—is the lack of granular data regarding the concurrent use of other glucose-lowering agents. In modern diabetes management, novel antidiabetic medications, particularly sodium-glucose cotransporter-2 inhibitors (SGLT2is) and glucagon-like peptide-1 receptor agonists (GLP-1 RAs), are frequently prescribed alongside metformin. These agents have demonstrated profound pleiotropic effects extending far beyond glycemic control, including robust cardiovascular protection, renal preservation, and systemic anti-inflammatory properties.

It is highly plausible that diabetic patients receiving metformin might also be on these newer agents as part of a combination therapy regimen. Consequently, the observed reduction in sepsis mortality and the improvement in end-organ function (such as the lower serum creatinine levels noted in our study) in the metformin cohort could be partially confounded by the unmeasured, synergistic benefits of these concurrent medications. Because the vast majority of the original primary studies neither reported comprehensive medication profiles nor statistically adjusted for these specific drug classes, we could not definitively isolate the independent survival benefit of metformin from potential combination therapies. Future large-scale prospective cohorts or individual patient data (IPD) meta-analyses that rigorously adjust for concurrent cardioprotective and renoprotective medications are essential to untangle these complex pharmacological interactions.

Finally, due to limited data, this meta-analysis evaluated only mortality as the clinical outcome measure. In subsequent research, our team will also document severity of illness scores (Acute Physiology and Chronic Health Evaluation II [APACHE II] and Sequential Organ Failure Assessment [SOFA] scores) before and after treatment, sepsis severity, requirements for mechanical ventilation, serum lactate levels, serum creatinine levels, ICU length of stay, and the occurrence of acute kidney injury at any stage. Comparisons will be made between groups to comprehensively evaluate the efficacy of preadmission metformin therapy on clinical outcomes in patients with sepsis with diabetes.

## Conclusion

This represents the most comprehensive meta-analysis to date investigating the association between metformin use and mortality in patients with sepsis with diabetes. The findings suggest that preadmission metformin use may be associated with reduced mortality in this population, including 28-day mortality, 90-day mortality, 365-day mortality, and in-hospital mortality. Clinically and from a public health standpoint, these data support the integration of metformin history as a favorable prognostic indicator into updated clinical guidelines, thereby informing future antimicrobial stewardship and sepsis bundle strategies. However, more robust support from additional high-quality studies is still warranted.

## List of abbreviations

ICUs, Intensive care units; mTOR, Mammalian target of rapamycin; PRISMA, Preferred Reporting Items for Systematic Reviews and Meta-Analyses; MOOSE, Meta-analysis of Observational Studies in Epidemiology; PROSPERO, International Prospective Register of Systematic Reviews; AMPK, Adenosine 5′-monophosphate (AMP)-activated protein kinase; CI, Confidence interval; NOS, Newcastle-Ottawa Scale; OR, Odds ratio; RC, Retrospective cohort; PO, Prospective observational study; M, Multiple; S, Single; MET, Metformin; NM, Non-metformin; NA, Not available; LA, Lactic acidosis.

## Data Availability

The original contributions presented in the study are included in the article/**Supplementary Material**. Further inquiries can be directed to the corresponding authors.
