# Supplementary material for: Association of preadmission metformin use and prognosis in patients with sepsis with diabetes: a systematic review and meta-analysis
Source: Front Endocrinol (Lausanne). 2026 Apr 20;17:1815219. doi: 10.3389/fendo.2026.1815219 (PMC13135973; doi:10.3389/fendo.2026.1815219)
Supplement: Supplementary file 1 [file DataSheet1.zip › Data Sheet 1/Supplemental Fig. 4.docx]

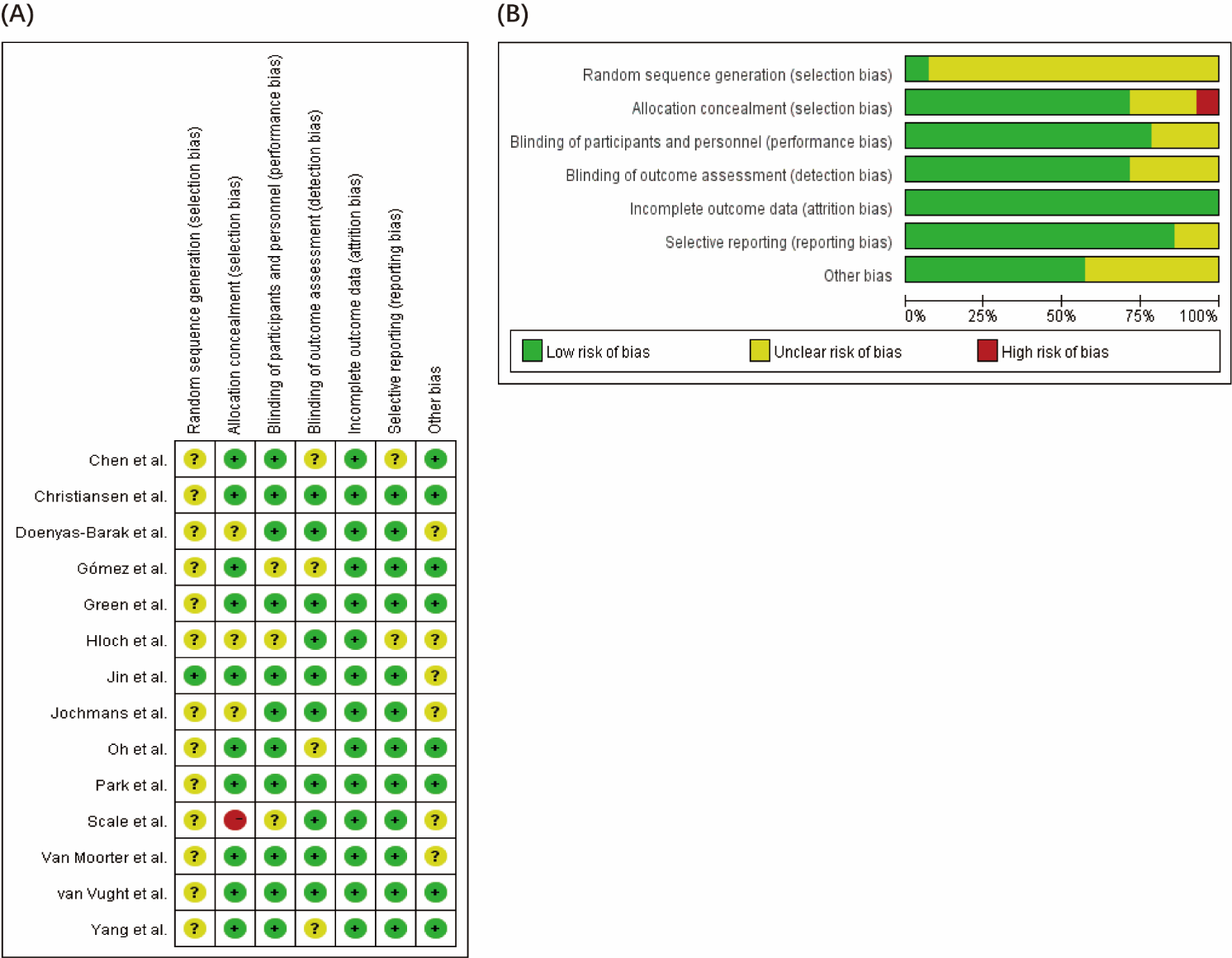


**Supplemental Fig. 4.** Risk of Bias in Non-Randomized Studies of Interventions tool with traffic lights (A). Risk of Bias in Non-Randomized Studies of Interventions tool with summary plot (B).
