# Supplementary material for: Association of preadmission metformin use and prognosis in patients with sepsis with diabetes: a systematic review and meta-analysis
Source: Front Endocrinol (Lausanne). 2026 Apr 20;17:1815219. doi: 10.3389/fendo.2026.1815219 (PMC13135973; doi:10.3389/fendo.2026.1815219)
Supplement: Supplementary file 1 [file DataSheet1.zip › Data Sheet 1/Supplemental Table 2.docx]

**Supplemental Table 2. The detailed search strategy**

| **Electronic databases** | **Search** | **Search strategy** | **Results** |
| --- | --- | --- | --- |
| **Pudmed** | #1 | (((sepsis[MeSH Terms]) OR (sepsis[Title/Abstract])) OR (Critically ill patients[MeSH Terms])) OR (Critically ill patients[Title/Abstract]) | 271,294 |
|  | #2 | (metformin[MeSH Terms]) OR (metformin[Title/Abstract]) | 35,016 |
|  | #3 | ((((sepsis[MeSH Terms]) OR (sepsis[Title/Abstract])) OR (Critically ill patients[MeSH Terms])) OR (Critically ill patients[Title/Abstract])) AND ((metformin[MeSH Terms]) OR (metformin[Title/Abstract])) | **216** |
| **EMBASE** | #1 | ('sepsis'/de OR sepsis:ab,ti OR 'critically ill patient':ab,ti OR 'critically ill patient'/de) | 379,192 |
|  | #2 | ('metformin'/de OR metformin:ab,ti) | 108,263 |
|  | #3 | #1 AND #2 | **1096** |
| **Cochrane CENTRAL** | #1  #2  #3  #4  #5  #6  #7  #8  #9 | (sepsis):ti,ab,kw  MeSH descriptor: [Sepsis] this term only  ("critically ill"):ti,ab,kw  MeSH descriptor: [Critical Illness] this term only  #1 OR #2 OR #3 OR #4  (metformin):ti,ab,kw  MeSH descriptor: [Metformin] this term only  #6 OR #7  #5 AND #8 | 14,946  3,168  13,445  3,864  26,875  14,448  5,498  14,448  **55** |
