# Supplementary material for: Association of preadmission metformin use and prognosis in patients with sepsis with diabetes: a systematic review and meta-analysis
Source: Front Endocrinol (Lausanne). 2026 Apr 20;17:1815219. doi: 10.3389/fendo.2026.1815219 (PMC13135973; doi:10.3389/fendo.2026.1815219)
Supplement: Supplementary file 1 [file DataSheet1.zip › Data Sheet 1/Supplemental Table 5.docx]

**Supplemental Table 5.** Summary of findings: Preadmission metformin use compared to no metformin use for sepsis patients with diabetes

| Outcomes | No. of studies (patients) | Study design | Risk of Bias | Inconsistency | Indirectness | Imprecision | Publication Bias | Upgrading Factors | Certainty of Evidence |
| --- | --- | --- | --- | --- | --- | --- | --- | --- | --- |
| 28-day mortality | 3 (3,855) | Observational^{a} | Not serious^{b} | Not serious^{c} | Not serious | Not serious^{d} | Not serious^{e} | None^{f} | ⊕⊕◯◯ LOW |
| 90-day mortality | 4 (3,299) | Observational^{a} | Not serious^{b} | Not serious^{c} | Not serious | Not serious^{d} | Not serious^{e} | None^{f} | ⊕⊕◯◯ LOW |
| 365-day mortality | 2 (367) | Observational^{a} | Not serious^{b} | Not serious^{c} | Not serious | Not serious^{d} | Not serious^{e} | None^{f} | ⊕⊕◯◯ LOW |
| In-hospital mortality | 4 (854) | Observational^{a} | Not serious^{b} | Not serious^{c} | Not serious | Not serious^{d} | Not serious^{e} | None^{f} | ⊕⊕◯◯ LOW |

**Explanations (Basis for downgrading/upgrading):**

a. Baseline Study Design: According to the GRADE framework, evidence derived exclusively from observational cohort studies intrinsically starts at a "Low" certainty of evidence level.

b. Risk of Bias: Assessed as "Not serious." All included observational studies achieved a Newcastle-Ottawa Scale (NOS) score of \ge 6, indicating a low risk of methodological bias in cohort selection and outcome assessment.

c. Inconsistency: Assessed as "Not serious." Although the overall pooled analysis showed moderate heterogeneity, stratifying the data by specific mortality timeframes (e.g., 28-day, 90-day) successfully reduced the I^2 statistics to acceptable levels within these specific subgroups.

d. Imprecision: Assessed as "Not serious." The pooled sample sizes within the subgroups were adequate, and the 95% Confidence Intervals (CIs) were relatively narrow and consistently remained below the clinical decision threshold (OR = 1.0).

e. Publication Bias: Assessed as "Not serious." Visual inspection of funnel plots, corroborated by quantitative Begg’s and Egger’s tests, detected no significant small-study effects or publication bias.

f. Upgrading Factors: Rated as "None." Although some outcomes demonstrated a relatively large magnitude of effect (e.g., OR < 0.5 for 90-day, 365-day, and in-hospital mortality), we conservatively chose not to upgrade the evidence to "Moderate". This decision was based on the substantial risk of unmeasured clinical confounding—such as the concurrent use of pleiotropic antidiabetic agents (e.g., SGLT2is) and variations in in-hospital medication continuation—which reduces absolute confidence in the magnitude of the independent effect estimate.
