# Supplementary figures and images for: Association of preadmission metformin use and prognosis in patients with sepsis with diabetes: a systematic review and meta-analysis
Source: Front Endocrinol (Lausanne). 2026 Apr 20;17:1815219. doi: 10.3389/fendo.2026.1815219 (PMC13135973; doi:10.3389/fendo.2026.1815219)

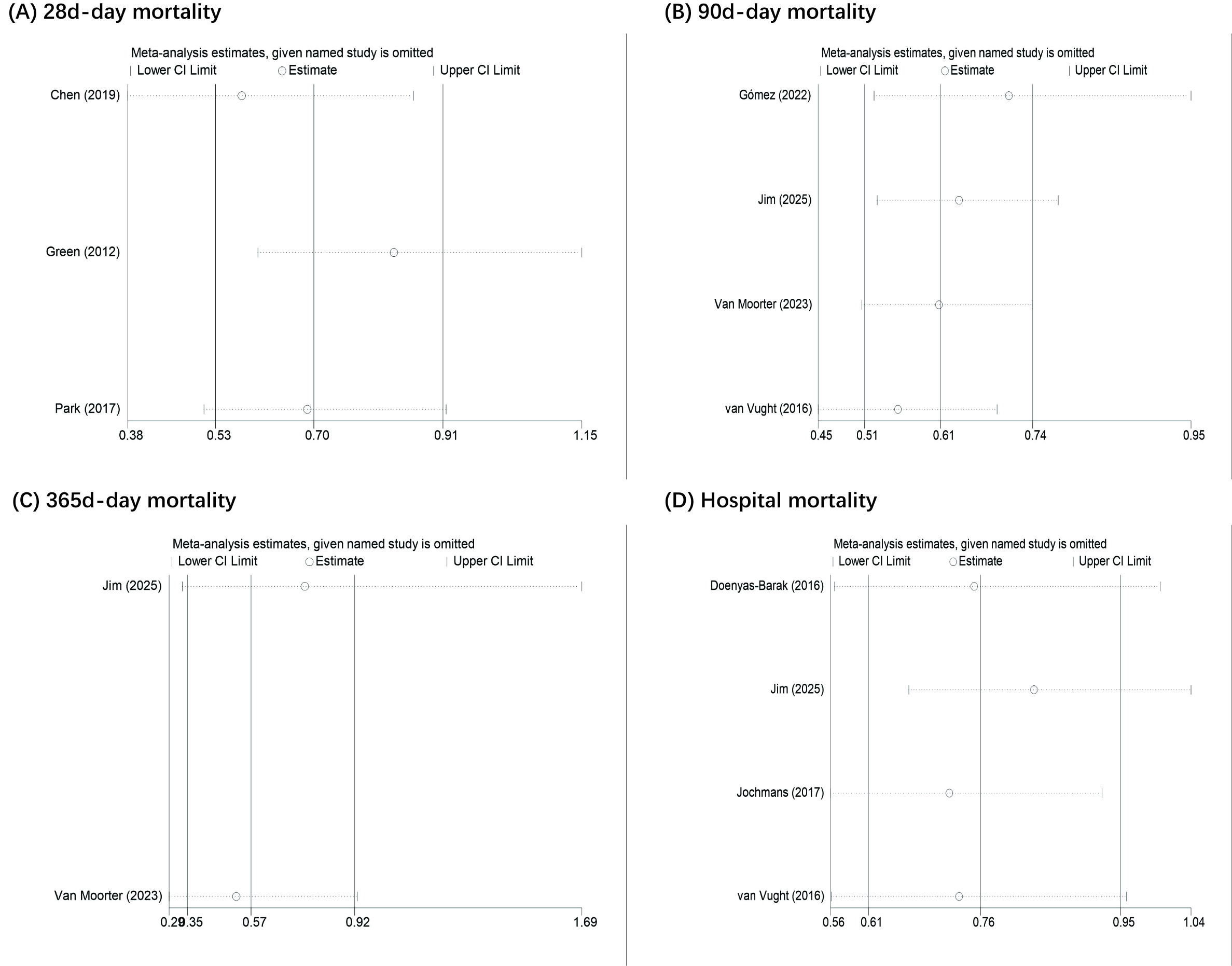

Supplement: Supplementary file 1 [file DataSheet1.zip › Data Sheet 1/Supplemental Fig. 1.tif]

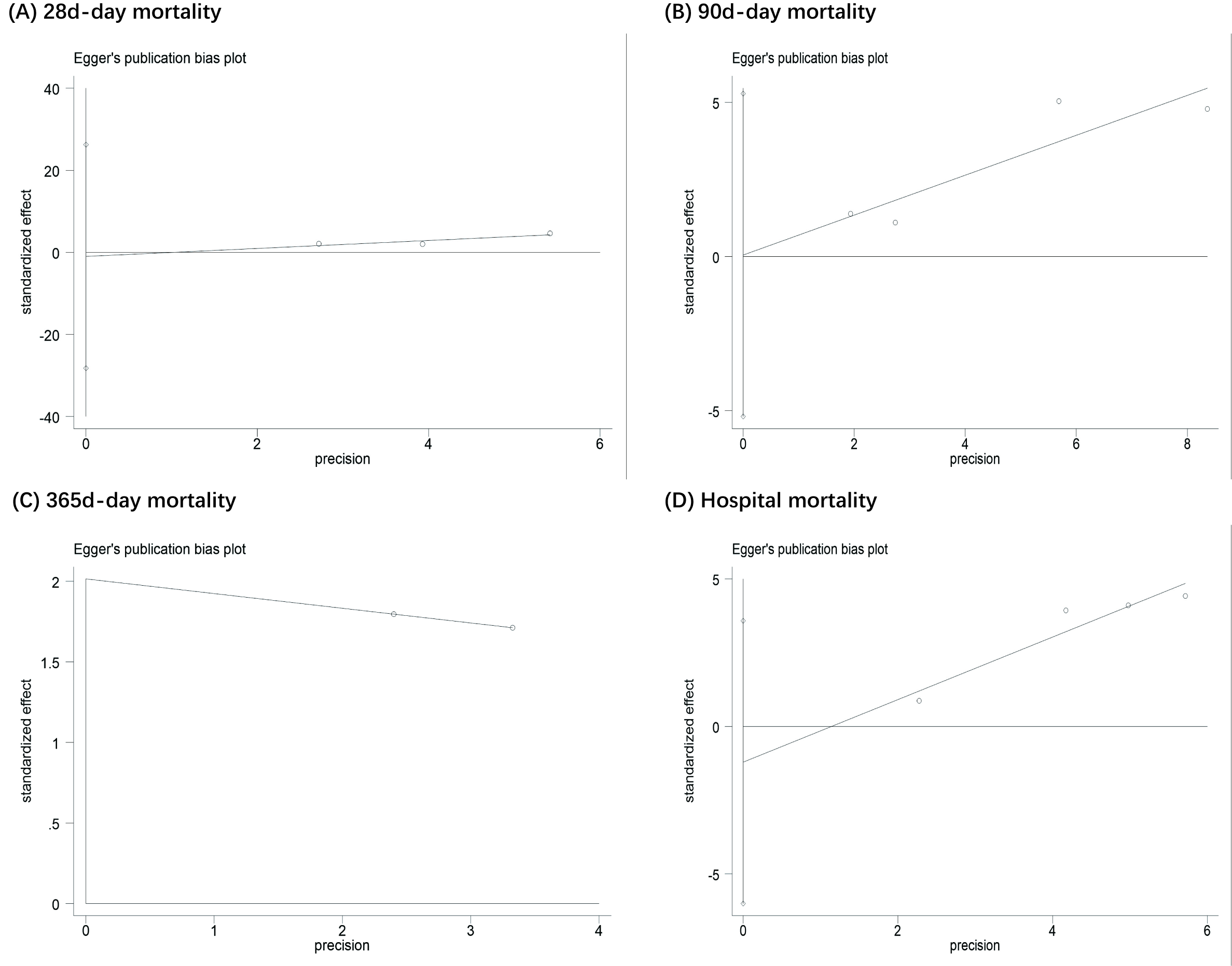

Supplement: Supplementary file 1 [file DataSheet1.zip › Data Sheet 1/Supplemental Fig. 2.tif]

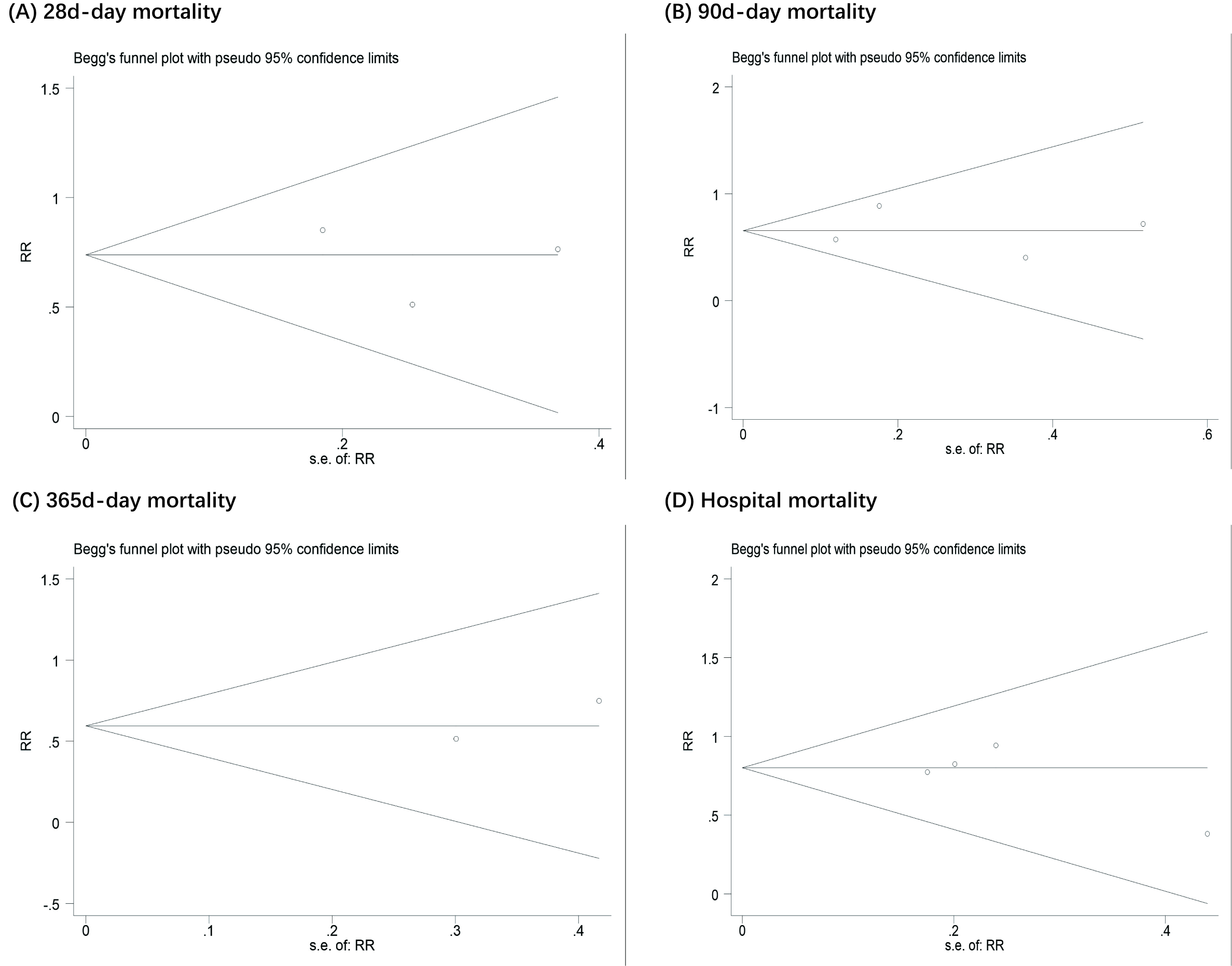

Supplement: Supplementary file 1 [file DataSheet1.zip › Data Sheet 1/Supplemental Fig. 3.tif]
